# Natural variation in stress response induced by low CO_2_ in *Arabidopsis thaliana*

**DOI:** 10.1515/biol-2020-0095

**Published:** 2020-12-21

**Authors:** Chunxia Wu, Yulou Sun, Guang Yang, Li Li, Wei Sun, Zenglan Wang, Hui Zhang, Yuanyuan Li

**Affiliations:** Shandong Provincial Key Laboratory of Plant Stress Research, College of Life Science, Shandong Normal University, Ji’nan, 250014, Shandong, People’s Republic of China; Key Laboratory of Systems Biology, College of Life Science, Shandong Normal University, Ji’nan, 250014, Shandong, People’s Republic of China

**Keywords:** CO_2_ limitation, *Arabidopsis*, plasticity, trait, slow growth

## Abstract

Variation in atmospheric carbon dioxide (CO_2_) concentration can dictate plant growth and development and shape plant evolution. For paired populations of 31 *Arabidopsis* accessions, respectively, grown under 100 or 380 ppm CO_2_, we compared phenotypic traits related to vegetative growth and flowering time. Four accessions showed the least variation in measured growth traits between 100 ppm CO_2_ and 380 ppm CO_2_ conditions, though all accessions exhibited a dwarf stature with reduced biomass under low CO_2_. Our comparison of accessions also incorporated the altitude (indicated in meters) above sea level at which they were originally collected. Notably, An-1 (50 m), Est (50 m), Ws-0 (150 m), and Ler-0 (600 m) showed the least differences (lower decrease or increase) between treatments in flowering time, rosette leaf number, specific leaf weight, stomatal density, and less negative δ^13^C values. When variations for all traits and seedset were considered together, Ws-0 exhibited the least change between treatments. Our results showed that physiological and phenotypic responses to low CO_2_ varied among these accessions and did not correlate linearly with altitude, thus suggesting that slower growth or smaller stature under ambient CO_2_ may potentially belie a fitness advantage for sustainable growth under low CO_2_ availability.

## 1 Introduction

Carbon dioxide (CO_2_) is a central and predominant environmental factor necessary for plant growth. As photosynthetic organisms, plants take up atmospheric CO_2_ by diffusion into the leaf through the stomata and subsequently convert it into organic compounds necessary for maintaining plant metabolism and sustained growth. Atmospheric CO_2_ concentration has varied tremendously throughout the history of plant life on Earth, ranging from as high as 3,000 ppm (parts per million) in the early Devonian (∼400 million years [myr] ago) [1] to as low as 180 ppm during the Pleistocene glacial (∼20 kilo-years [kyr] ago) [2]. Variation in CO_2_ has been proposed as a driver of plant evolution [3,4]. Substantial previous research has established that elevated concentrations of atmospheric CO_2_ can exert clear phenotypic effects on plants such as increased photosynthetic rates, which in turn lead to higher crop yields and reduced water loss by transpiration [5,6,7,8]. Falling global atmospheric CO_2_ potentially imposes a selective pressure on vascular plants that can drive evolutionary trajectories for increased stomatal density (SD), decreased individual stomatal size [9,10], higher vein density, and greater water-use efficiency [11,12]. Several studies have thus postulated that around 30 myr ago, an abrupt drop in atmospheric CO_2_ induced the emergence of C_4_ species [13,14,15,16,17].

Previous studies have proposed that modern C_3_ plants experience heightened stress under low CO_2_ and may respond by changing their reproductive or developmental timing or by changing their allocation of biomass to different tissues, resulting in measurable, phenotypic responses to low CO_2_ that may be potentially inherited if the environmental conditions persist [18]. For example, Billings et al. [19] observed adaptive variation in *Oxyria digyna*, in which high-altitude ecotypes were capable of higher photosynthetic rates and lower CO_2_ compensation points compared to low-altitude ecotypes across a range of CO_2_ concentrations, including low CO_2_.

*Arabidopsis* (*Arabidopsis thaliana* (L.) Heynh) is widely distributed throughout the Northern Hemisphere and adapts to a broad range of climatic conditions and selective pressures [20,21]. Sharma et al. [22] grew 33 *Arabidopsis* accessions below the compensation point (achieved by growing the C_4_-plant maize alongside *Arabidopsis*) and found a difference of over 1 week in survival time among accessions. Ward and Strain [23] showed that *Arabidopsis* accessions from different elevations had significant variation in seed yield when grown at low CO_2_ (200 ppm). Ward and Kelly [24] observed a high level of genetic variation in percentage survival, reproductive output, and total seed production among the *Arabidopsis* genotypes when grown at low CO_2_ (200 ppm). Taken together, these studies suggest that *Arabidopsis* has phenotypic plasticity in response to low CO_2_, and natural accessions of *Arabidopsis* can vary widely genetically and phenotypically for many traits [20,25].

In order to survive and successfully reproduce in a given environment, plants must fix carbon to produce biomass, then initiate and complete their reproductive stage, in which plants direct energy into flowering and seed production. Several traits related to C3 and C4 carbon metabolism are essential for developing sufficient biomass for the plant to adequately support the production of flowers and seeds. For example, the trait of flowering time is critically important for reproductive fitness since plants must find pollinators (i.e., flowers of the same species) for successful outcrossing [26]. Similarly, the timing of seedset is extremely important for ensuring that seed is dispersed into conducive environmental conditions among selfing species [26]. Furthermore, these traits are regulated by external, environmental signals as well as internal, physiological cues [26].

Low CO_2_ has been shown to induce molecular changes in addition to a variety of phenotypical trait changes in *A. thaliana*. Growth on petri dishes wrapped with Parafilm led to CO_2_ deprivation as soon as cotyledons emerged [27]. This CO_2_ deprivation resulted in a 35% difference in the expression of biochemical pathways, such as those for carbohydrate metabolism, chlorophyll biosynthesis, secondary metabolite biosynthesis, and stress response, compared with fully aerated plants [27]. Specifically, short-term CO_2_ limitation (an 8 h shift from 10,000 ppm CO_2_ to 380 ppm CO_2_) did not cause visible changes in phenotype but significantly induced transcriptional and metabolic responses in five genes related to photorespiration through glycerate, glycolate, serine, and glycine production [28]. Moreover, when 5-week-old *Arabidopsis* plants were transferred into 100 ppm CO_2_ conditions for 24 h, ornithine accumulated, which is an intermediate of the urea cycle and a central metabolite of arginine synthesis and degradation [29]. Long-term low CO_2_ stress was induced in *Arabidopsis* Col-0 by growth in 100 ppm CO_2_ for 6 weeks [30]. The genes upregulated at 100 ppm CO_2_ were remarkably enriched in stress response and the downregulated genes were only significantly enriched in cell wall and endomembrane system [31]. However, energy metabolism, lipid metabolism, and amino acid metabolism pathways showed significant decreases in flux under low CO_2_, whereas nucleotide metabolism showed increased flux [31].

For these reasons, in this study, we chose to focus on flowering time, seedset, and several marker traits at flowering time, including aboveground biomass, rosette leaf number, SD, specific leaf weight (SLW), and stable isotope carbon assimilation as metrics for the ability to adapt to low CO_2_ among different wild *Arabidopsis* accessions. We hypothesized that accessions capable of adaptation to growth under low CO_2_ would show the least variation in biomass production, carbon assimilation, and flowering time compared to their growth under ambient CO_2_, whereas plants lacking the genetic variation that allows adaptation to low CO_2_ cannot successfully grow or reproduce under carbon-limited conditions. We thus compared growth during the vegetative and reproductive development of 31 *Arabidopsis* accessions under low CO_2_ (100 ppm) and ambient CO_2_ (380 ppm), to better understand the contribution of natural, heritable variation to the plant response to low CO_2_. This work contributes to the findings of previous studies that explored the genetic variation underlying evolutionary adaptations such as C4 metabolism, while also providing meaningful context for observable changes in wild populations that are subject to current changes in climate and atmospheric CO_2_.

## 2 Materials and methods

### 2.1 Plant materials and growth conditions

Thirty-one *A. thaliana* accessions were used in this study (Table 1) to represent a wide range of geographically separated locations, elevations, and climates.

**Table 1:** Accessions used in this study and their locations of origin and altitudes in meters

Accessions	Stock number	Country	Location	Altitude (m above sea level)
Col-0	CS1092	USA	Columbia	50
An-1	CS6603	Belgium	Antwerpen	50
Ct-1	CS6674	Italy	Catania	50
Est	CS6173	Germany		50
Lc-0	CS6769	UK	Loch Ness	50
Litva	CS925	Lithuania		50
Lm-2	CS6784	France	Le Mans	50
Pa-1	N1439	Italy	Palermo	50
Per-1	CS1444	Russia	Perm	50
Ren-1	CS22253	France	Rennes	42
Te-0	CS6918	Finland	Tenela	50
Ts-1	A22647	Spain	Tossa del Mar	50
Tsu-1	CS6926	Japan	Tsushima	50
Van-0	CS6884	Canada	University of British Columbia	50
Wt-5	CS6896	Germany	Wietze	50
Be-0	CS6613	Germany	Bensheim/Bergstr.	150
Ga-0	CS1181	Gabelstein	Gabelstein	150
Mt-0	CS6799	Libya	Martuba/Cyrenaica	150
Rsch-4	CS1494	Russia	Rschew/Starize	150
Stw-0	CS6865	Russia	Stobowa/Orel	150
Ws-0		Russia	Wassilewskija	150
Kin-0	CS1272	USA	Kindalville, MI	300
Bay-0	CS6608	Germany	Bayreuth	350
Bs-1	CS6627	Switzerland	Basel	350
Kil-0	CS6754	UK	Killean	450
Lip-0	CS1336	Poland	Lipowiec/Chrzanow	500
Ler-0	CS163	Germany		600
Mc-0	CS1363	UK	Mickle Fell	700
Ka-0	CS6752	Austria	Karnten	950
Kas-1	CS903	India	Kashmir	1,580
Sha		Tadjikistan	Pamiro-Alay	3,400

Note: Stock number (N, NASC stock center (http://arabidopsis.info/); A, ABRC stock center (http://abrc.osu.edu/)).

*Arabidopsis* seeds were surface-sterilized by soaking in 75% (v/v) ethanol for 10 min and rinsed 5–6 times with 95% ethanol, then sown on solid media containing half-strength Murashige and Skoog mineral salts, 1% (w/v) sucrose, and 0.8% (w/v) agar, pH 5.7. Plates with seeds were incubated in the dark at 4°C for 2 days to break dormancy prior to germination in growth chambers. The 7-day-old seedlings were transferred to a mixture of perlite/vermiculite/peat (1:1:3) in a 8 cm square pot (512 cm^3^). For each CO_2_ condition, at least 50 seedlings for one accession were transferred into the pot. Plants were then grown in a Percival controlled environment (E-36L, USA) growth chamber either at low CO_2_ (100 ppm) or ambient CO_2_ (380 ppm) with a 16 h light (22°C)/8 h dark (18°C) photoperiod and a light intensity of 120 µmol m^−2^ s^−1^ and 70% humidity. The CO_2_ concentration was set the same as our previous study [30]. Four chambers, two for low CO_2_ and the other two for 380 ppm CO_2_, were used. The plants in the two (under the same condition) chambers were switched twice a week.

### 2.2 Growth parameters

Boyes et al. [32] defined 30 growth stages, which were divided into 9 principal stages for *Arabidopsis*, spanning development from seed imbibition through the completion of flowering and seed maturation. Based on the physiological growth stages of *A. thaliana* established by Boyes et al. [32], we chose stage 5.10 (first flower buds visible) and stage 6.00 (first flower open) to measure the growth parameters. At the beginning of stage 5.10, the transition from vegetative growth to reproductive growth, we recorded the number of days since germination until the first flower buds were visible, as well as the aboveground fresh weight (FW), number of rosette leaves, SLW, and the δ^13^C value in leaves. At the beginning of stage 6.00, we again recorded the number of days between germination and the opening of the first flower, as well as SD. Individual leaves were detached from each plant with forceps and imaged for subsequent analysis using a scanner (V900; Shanghai MICROTEK Technology Co. Ltd, Shanghai, China). Length and area were measured using IMAGE J (v1.8.0, https://imagej.nih.gov/ij/index.html) software.

### 2.3 Stable carbon isotope analysis

The fully expanded third true leaf of each plant that developed before stage 5.10 was used to quantify the stable carbon isotope ratio (^13^C/^12^C). All samples were oven-dried at 65°C for 48 h to a constant weight. The measurements of stable carbon isotope ratios were carried out at the Chinese Academy of Forestry’s Stable Isotope Laboratory (Beijing, China) using a Flash EA1112 HT elemental analyzer (Thermo Fisher Scientific, Waltham, MA, USA) coupled with a Delta V advantage isotope ratio mass spectrometer (Thermo Fisher Scientific). Stable carbon isotope ratios were expressed as δ^13^C (‰) and were calculated as follows:

{\delta }^{13}\text{C}(\textperthousand )=\left[\left({R}_{\text{sample}}/{R}_{\text{standard}}\right)-1\right]\times 1,\hspace{-0.25em}000,

where *R*_sample_ and *R*_standard_ are the ratios of ^13^C/^12^C in the samples and the standard (Pee Dee Belemnite), respectively. The precision of the repeated sample was 0.15‰.

### 2.4 SD measurement

The largest, fully expanded leaves were selected for SD measurement and prepared as follows: (1) leaves were fixed overnight or longer in FAA solution (5 mL of formaldehyde:5 mL of acetic acid:90 mL of 70% ethanol); (2) leaves were decolorized in 70% ethanol until white; (3) tissue samples were mounted abaxially on slides with Hoyer’s solution; and (4) stomata were visualized by differential interference contrast microscopy on a Zeiss Imager Z2 microscope (Carl Zeiss Microscopy, LLC, White Plains, NY, USA) (0.379 mm^2^ field of view). Ten images were collected from the middle of the abaxial side of each leaf sample, between the mid-vein and the edge. Stomata were manually counted for all pictures and all leaves using IMAGE J (v1.8.0, https://imagej.nih.gov/ij/index.html). Six leaves per accession were analyzed.

### 2.5 Statistical analysis

Statistical analyses for all experiments were performed using Excel 2010 (Los Angeles, CA, USA), SPSS 19.0 (SPSS Inc., Chicago, IL, USA), and SigmaPlot (SyStat Software, San Jose, CA, USA) software. After calculating averages, standard deviations and standard errors were also determined. Significant differences between low and ambient CO_2_ treatments for each trait and the interaction effect of CO_2_ and accessions were determined by one-way analysis of variance with *p* ≤ 0.05 for each experiment.

## 3 Results

### 3.1 Effect of treatment on traits

**Figure A1:**
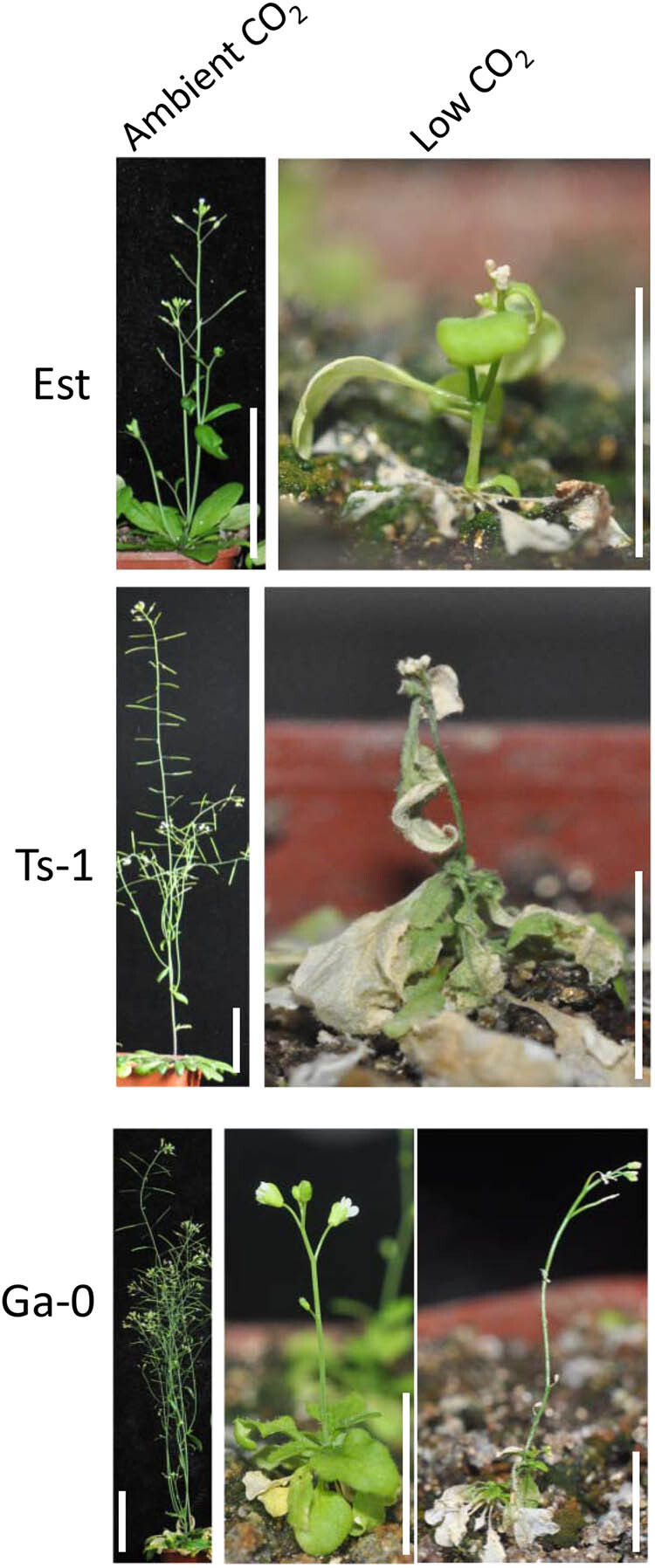
Three accessions died under low (100 ppm) CO_2_. Est and Ga-0 accessions died after stage 6.00 when grown in low CO_2_. The first flower of Est partly opened but withered gradually and died; a portion of the Ga-0 flower buds opened but had no seed in siliques and died under low CO_2_. The flower buds of Ts-1 failed to open under low CO_2_ conditions. The scale bars in images of flower buds grown in ambient CO_2_ indicate 5 cm and those in low CO_2_ indicate 1 cm.

In this experiment, the low CO_2_ concentration was set to 100 ppm, which was shown to be a severe stress to *Arabidopsis* ecotype Columbia-0, and the ambient CO_2_ was set to 380 ppm the same as those in our previous study [30]. A collection of 31 accessions (Table 1) was selected to analyze the genetic diversity based on the whole set of measurable responses to low CO_2_. As expected, all tested accessions showed reduced growth when grown under low CO_2_ versus ambient CO_2_ (380 ppm) (Data not shown. Part results are shown in Figure A1).

As shown in Table 2, the effects of CO_2_ concentration and accession were strongly significant in the comparison of the number of days to stage 5.10, FW, number of rosette leaves, SLW, and SD. The interaction effect of CO_2_ and accession on these five traits was also highly significant.

**Table 2:** Effects of CO_2_, accession, and CO_2_ × accession interaction for days to stage 5.10, FW, number of rosette leaves, SLW, and SD (*F* values are shown)

Variation source	Days to stage 5.10	FW	No. of rosette leaves	SLW	SD
CO_2_	1,192^***^	3,494^***^	93^***^	6,978^***^	37^***^
Accession	1,336^***^	180^***^	192^***^	47^***^	132^***^
CO_2_ × Accession	388^***^	139^***^	83^***^	49^***^	34^***^

Note: the significance level: *p < 0.05; **p < 0.01; ***p < 0.001.

### 3.2 Variation in flowering time

**Figure 1:**
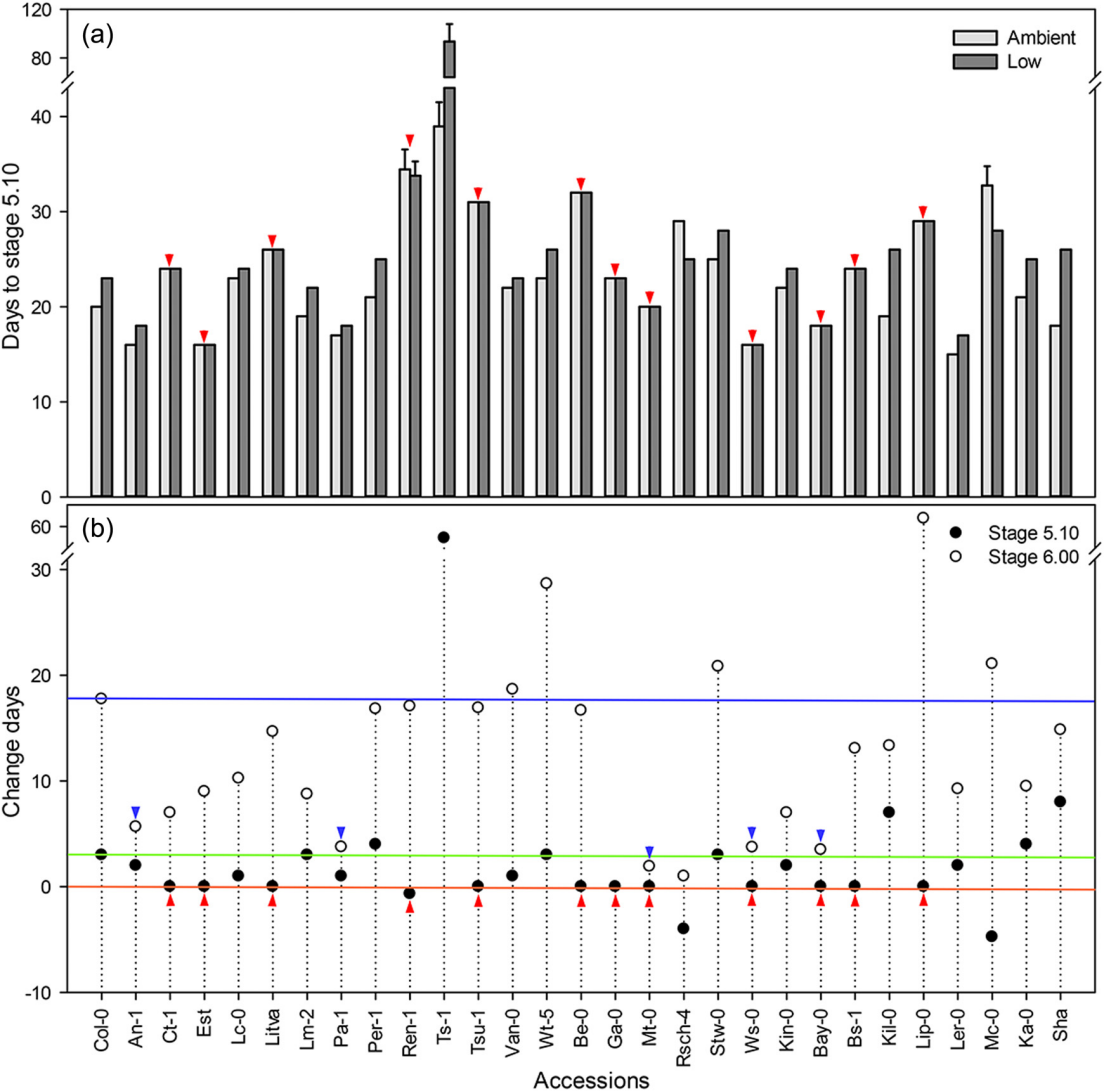
Effect of low CO_2_ on flowering time. (a) Days from germination to stage 5.10. (b) The difference in time from germination to stage 5.10 or 6.00 for plants grown under low CO_2_ compared with those under ambient CO_2_. Values are mean ± SE (*n* = 3). Red arrows indicate the accessions with no difference in duration from germination to stage 5.10 between the two CO_2_ treatments. Blue arrows indicate the accessions exhibiting a shorter time in days to reach stage 6.00 between the two CO_2_ treatments. *Arabidopsis* accessions listed on the *x*-axis (left to right) are arranged by altitude, in the same order as in Table 1.

The onset of flowering, which is the transition from vegetative to reproductive stages, is a major determinant of a plant’s reproductive success and may be hastened or delayed by variations in climate that act as environmental cues or stimuli for the plant [33]. We measured the time from germination to the appearance of the first visible flower bud (developmental stage 5.10 as described in [32]) and the time to the first flower opening (developmental stage 6.00; [32]) of 31 accessions grown under low CO_2_ and ambient CO_2_, and calculated the difference in flowering times between the two CO_2_ treatments. Two accessions, Mc-0 and Rsch-4, made the transition to flowering (stage 5.10) 4 days earlier under low CO_2_ than under ambient CO_2_ (Figure 1a and b). In 17 accessions, the low CO_2_ treatment delayed flowering (stage 5.10) for at least 1 day. Among these, Ts-1 took 54 days longer to reach stage 5.10 under low CO_2_ (Figure 1a and b). Te-0 and Kas-1 never flowered and died under low CO_2_, so the data from these two accessions were missing in the following analysis. Twelve accessions (Figure 1b, red arrows) showed no difference in the time to stage 5.10 under low CO_2_ and ambient CO_2_, including Est, Ws-0, and Ler-0.

The time to the appearance of an open flower (stage 6.00) was far more variable than the time to stage 5.10, even though the timing of flower opening was consistently delayed in all the accessions when grown under low CO_2_ (Figure 1b). This delay in the first flower opening ranged from 1 day (Rsch-4) to 63 days (Lip-0). Five accessions, including An-1, Pa-1, Mt-0, Ws-0, and Bay-0, showed less difference between the time to stage 6.00 under low CO_2_ and ambient CO_2_ (Figure 1b). Two accessions Est and Ga-0 died after reaching stage 6.00 in low CO_2_ conditions. Under low CO_2_, the first flower of Est opened partly but withered gradually and died, while a portion of the Ga-0 flower buds opened but had no seed in siliques and also subsequently died. The flower buds of Ts-1 failed to open under low CO_2_ condition (Figure A1). Given the importance of a consistent flowering time when all conditions are stable except CO_2_, we postulated that accessions that were able to maintain their time of flowering in spite of low CO_2_ exhibited higher adaptability than accessions with a greater difference in flowering time. Supporting this point, five accessions failed to flower successfully and died at stages 5.10 and 6.00, indicating that they were unable to pass this developmental stage under low CO_2_.

### 3.3 Aboveground biomass

**Figure 2:**
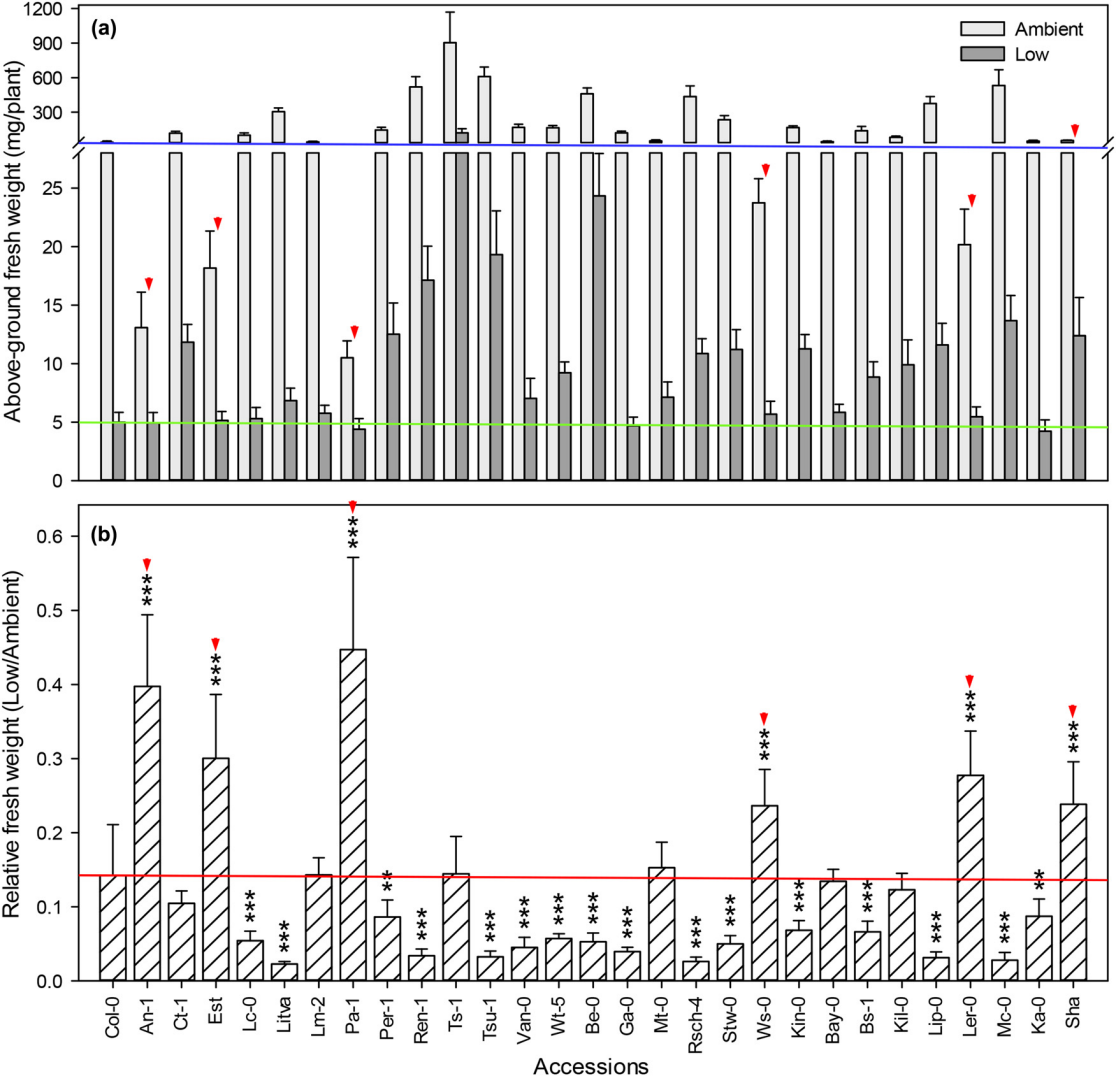
Effect of low CO_2_ on shoot biomass. Relative FW, the ratio of FW under low CO_2_ to FW under ambient CO_2_. Red arrows indicate the accessions screened out by shoot biomass. Values are mean ± SE (*n* = 3). Stars denote significant differences between Col-0 and other accessions (*p < 0.05, ***p < 0.001).

Biomass is frequently used as a reliable estimate of plant fitness [34]. All the accessions tested in this study exhibited a reduction in plant size during low CO_2_ growth. We measured aboveground biomass at the time of flowering (stage 5.10) and found that the aboveground (shoot) FW of all accessions decreased significantly (*p* < 0.001) under low CO_2_ compared to biomass of plants grown under ambient CO_2_ (Figure 2a). Two accessions, Pa-1 and An-1, showed a 60% reduction and four accessions showed a 70–80% reduction in shoot FW. The percent decrease for 7 accessions was between 80 and 90%, and for 16 accessions, biomass decreased over 90% (Figure 2a).

We also calculated the variation in relative FWs between the two treatments by determining the ratio of shoot FW under low CO_2_ to normal CO_2_. We found that compared to Col-0, the accessions An-1, Est, Pa-1, Ws-0, Ler-0, and Sha all showed lower variation in relative FW when grown in CO_2_-limiting conditions (Figure 2b). As with flowering time, we considered lower variation in FW for plants grown under low CO_2_ compared to ambient CO_2_ to be an indicator of higher adaptability by these accessions.

### 3.4 Rosette leaf number

**Figure 3:**
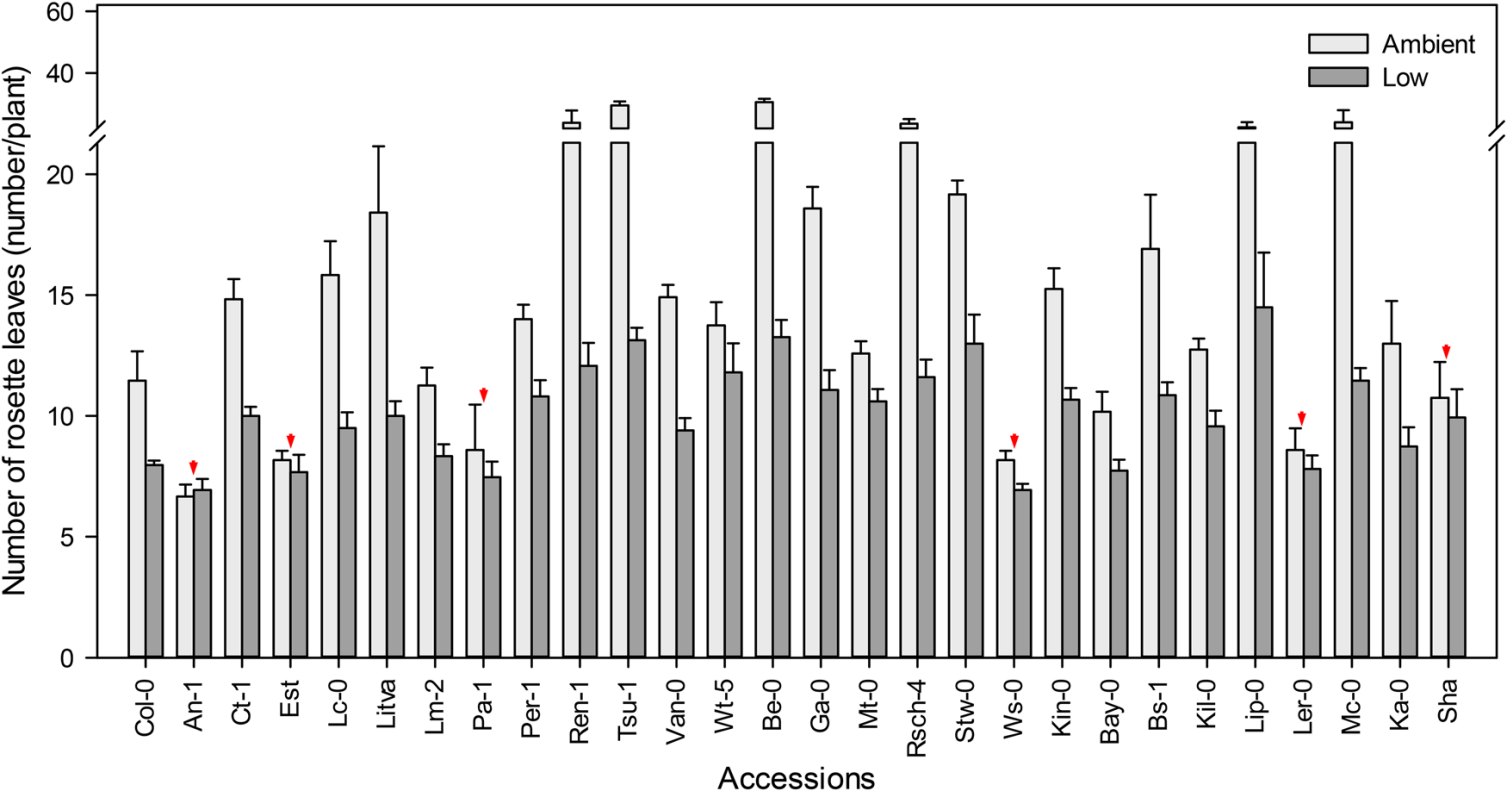
Effect of low CO_2_ on rosette leaf number. Red arrows indicate the accessions with the least difference in leaf number between the two CO_2_ treatments. Values are mean ± SE (*n* = 3).

The leaf number is closely correlated with the time to flowering and can be used as an indicator of phenotypic variability among different *Arabidopsis* accessions [35]. We counted the number of leaves in the rosette (excluding cotyledons) at the time of the first visible flower bud. Under low CO_2_, most of the accessions bolted, resulting in fewer rosette leaves. For example, Be-0, Tsu-1, Mc-0, and Rsch-4 exhibited a greater than 50% reduction in leaves compared to those growth in ambient CO_2_. However, An-1, Est, Pa-1, Ws-0, Ler-0, and Sha showed only a slight difference in rosette leaf number between treatments. Specifically, the leaf number of An-1 in low CO_2_ was slightly greater than under ambient CO_2_, whereas the other five accessions had on average one leaf less when grown under low CO_2_ (Figure 3).

We also counted the number of cauline leaves present at the time of the first flower opening. The cauline leaf response to low CO_2_ was more variable among accessions than the rosette leaf response. On average, the number of cauline leaves was reduced under low CO_2_ (data not shown), although in contrast, Wt-5 and Lip-0 had more cauline leaves due to the longer developmental time prior to reaching stage 6.00 from stage 5.10 under low CO_2_. These two accessions also had more lateral branches.

Interestingly, there were four accessions for which the number of cauline leaves was less than 20% higher in low CO_2_, whereas An-1 increased by 60% in low CO_2_ compared to plants grown without CO_2_ limitation. In contrast, 12 accessions exhibited a reduction in cauline leaves of less than 20% under low CO_2_ and 9 accessions had 20–66% fewer leaves under CO_2_-limiting treatment. The An-1, Est, Pa-1, Bay-0, Sha, and Wt-5 accessions had less than two leaves under ambient CO_2_, resulting in percent difference of less than 20% except for An-1 and Wt-5. Since the role of cauline leaves in photosynthetic productivity is less certain than for rosette leaves given their typical variability under unmodified atmospheric CO_2_, the contribution of variability in production of these leaves is also less predictable than that of rosette leaves, though in either case, we hypothesize that low variability indicates higher adaptability to low CO_2_.

### 3.5 SD

**Figure 4:**
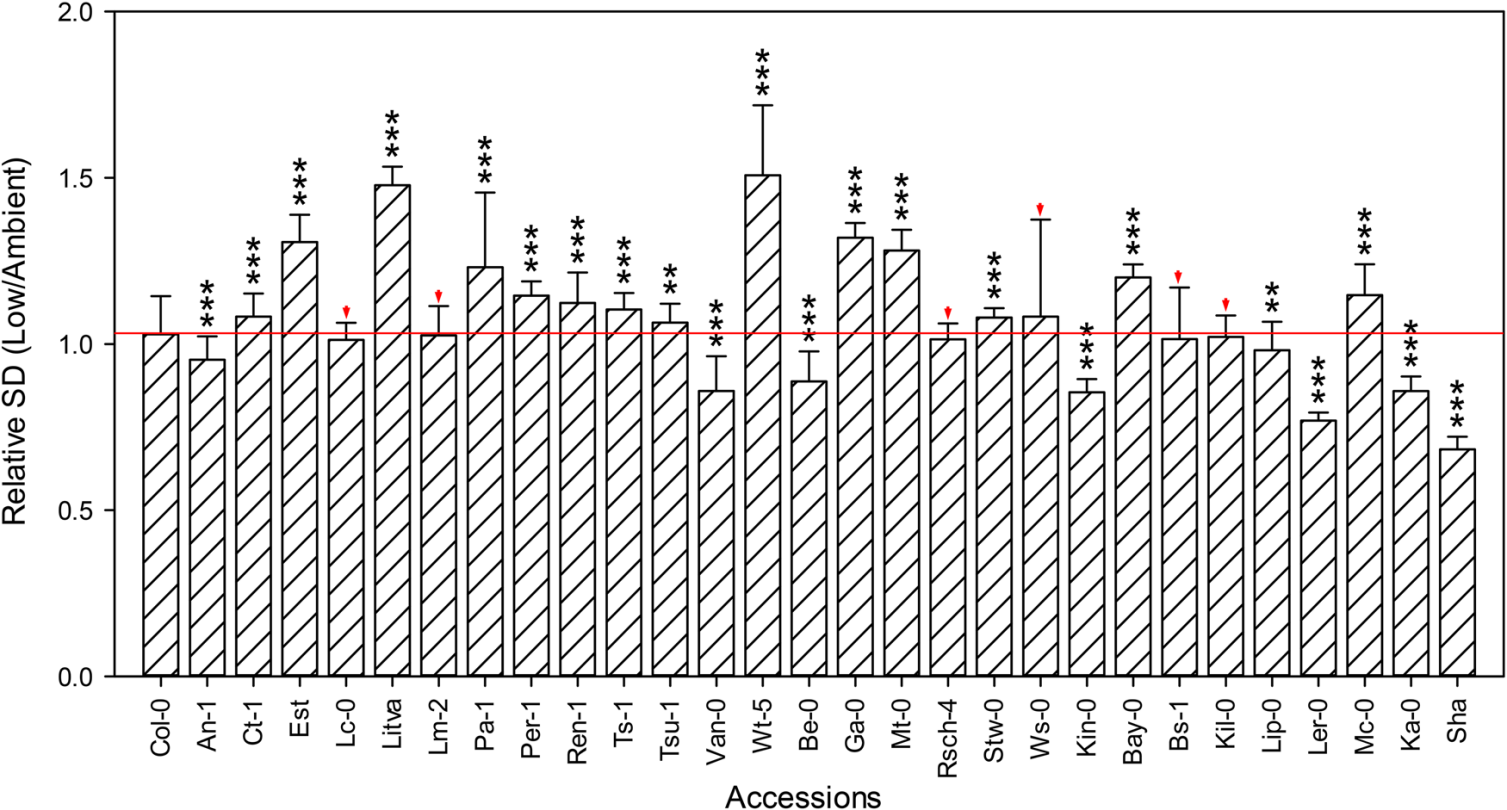
Effect of low CO_2_ on SD. Relative SD is the ratio of the SD under low CO_2_ to the SD under ambient CO_2_. Red arrows indicate the accessions with no significant difference in relative SD compared to Col-0. Values are mean ± SE (*n* = 3). Stars denote significant differences between Col-0 and other accessions (*p < 0.05, ***p < 0.001).

Stomata are present on the leaf surface and control the entry of CO_2_ into the leaves of plants prior to assimilation via photosynthesis [36,37,38]. Previous studies have reported that in *Arabidopsis* ecotype Col-0, SD (the number of stomata per unit leaf area) increased in response to low CO_2_ concentration [30]. Here, we examined the SD of the abaxial (lower) leaf blade epidermis of the surviving *Arabidopsis* accessions grown under ambient and low CO_2_ conditions. Among these 29 *Arabidopsis* accessions, there were 14 whose SD was significantly higher under low CO_2_ compared to ambient CO_2_ (Figure 4). Lc-0, Lm-2. Rsch-4, Ws-0, Bs-1, and Kil-0 did not show any significant differences in SD compared with Col-0. However, the SD of several accessions, including An-1 and Ler-0, decreased in response to low CO_2_ (Figure 4). We are inclined to speculates, in light of these results, that accessions showing increased SD have higher fitness under low CO_2_, since the higher number of stomata can increase the rate of CO_2_ diffusion in leaves.

### 3.6 SLW

**Figure 5:**
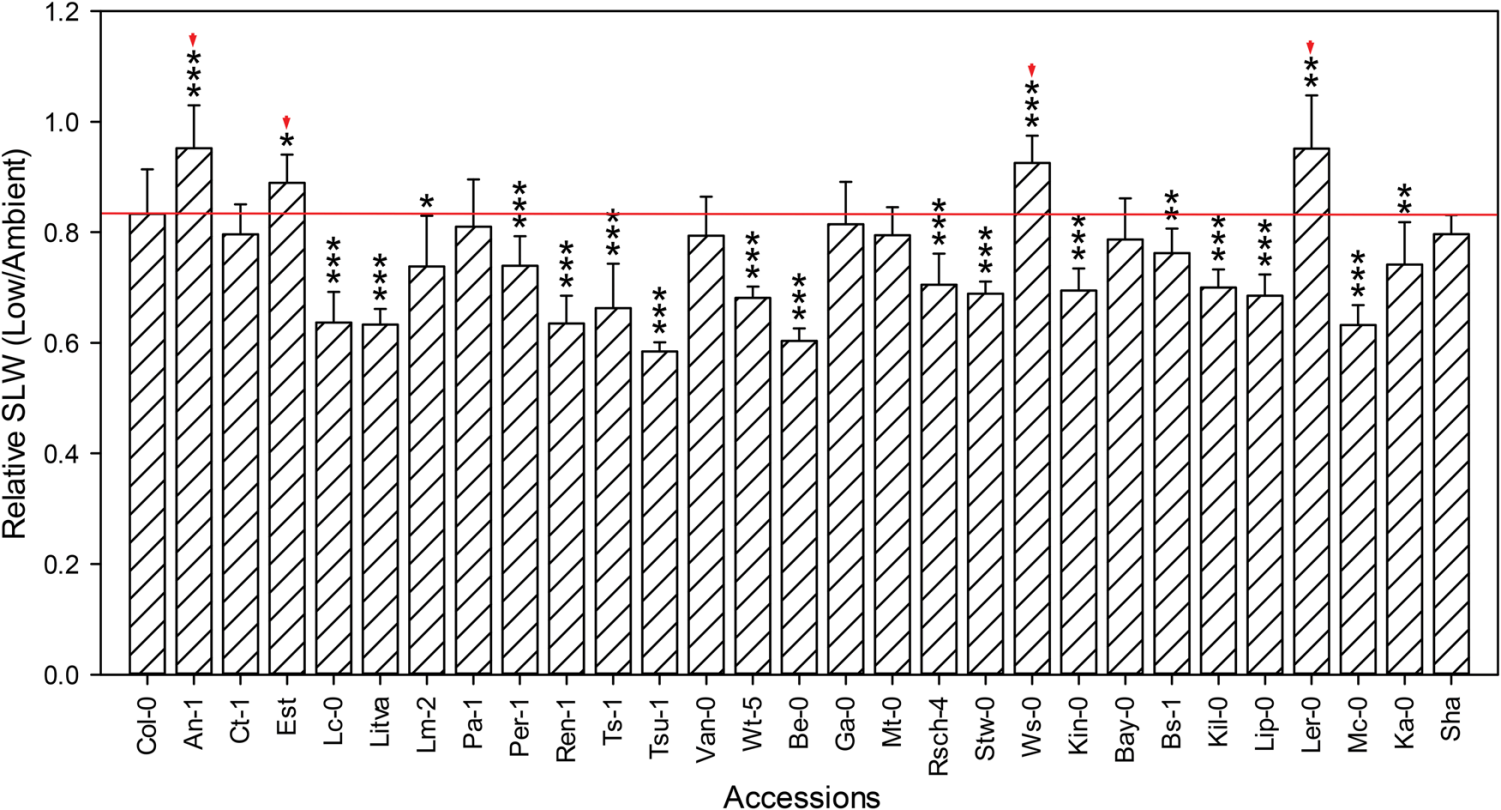
Effect of low CO_2_ on SLW of accessions. Relative SLW is the ratio of the SLW under low CO_2_ to the SLW under ambient CO_2_. Red line delineates the relative SLW of Col-0. Red arrows indicate the accessions with higher relative SLW compared to Col-0. Values are mean ± SE (*n* = 3). Stars denote significant differences between Col-0 and the other accession (*p < 0.05, ***p < 0.001).

SLW is defined as unit weight per unit leaf area, and it is an indicator of plant photosynthetic capacity, with high SLW interpreted as a decrease in photosynthetic efficiency [39,40,41,42]. In general, at low CO_2_, SLW was lower than at ambient CO_2_ among the *Arabidopsis* accessions in this screen. Here, we used the relative SLW, or the ratio of SLW under low CO_2_/SLW under ambient CO_2_, to evaluate the photosynthetic adaptations in response to low CO_2_. Compared to Col-0, the accessions An-1, Est, Ws-0, and Ler-0 showed substantially lower variation between two treatments (Figure 5).

### 3.7 Stable carbon isotope ratio of leaf tissue (δ^13^C)

The stable carbon isotope ratio is used to distinguish the photosynthetic CO_2_-fixing pathway in plants [43,44]. The δ^13^C values of C3 plants are typically more negative than those of C4 plants (−23 to −32% vs −6 to −19%, respectively) [43,44]. However, *Arabidopsis* carries some genes that belong to the C4 pathway, leading us to hypothesize that under ambient CO_2_, this species may exhibit a less negative δ^13^C value under low CO_2_ than ambient CO_2_. To investigate the effect of low CO_2_ on the photosynthetic capability of *Arabidopsis* accessions, we measured δ^13^C values in the third true leaves of all accessions, in order to analyze the stable carbon isotope ratio during treatment with low CO_2_. Unexpectedly, the δ^13^C values of seven accessions at low CO_2_ were more negative compared to those at ambient CO_2_, whereas most of the other accessions had more positive δ^13^C values under low CO_2_ treatment (Table 3), including the accessions An-1, Est, Ws-0, and Ler-0, thus suggesting a potential role for C4 genes in future potential adaptations to low CO_2_.

**Table 3:** Mean δ^13^C value of *Arabidopsis* accessions (*n* = 3)

Accessions	Ambient (380 ppm) CO_2_ (‰)	Low (100 ppm) CO_2_ (‰)	L-A (‰)
Ga-0	−32.95	−35.8	−2.86
Be-0	−33.33	−34.48	−1.16
Tsu-1	−33.16	−33.82	−0.66
Bs-1	−34.37	−34.77	−0.41
Wt-5	−33.26	−33.47	−0.2
Ct-1	−36.96	−37.13	−0.17
Stw-0	−33.99	−34.04	−0.06
Kin-0	−36.25	−35.91	0.34
Lip-0	−33.01	−32.65	0.36
Mt-0	−38.14	−37.64	0.5
Mc-0	−33.93	−33.25	0.68
Ren-1	−33.39	−32.61	0.78
Litva	−33.25	−32.23	1.03
Est	−39.37	−38.34	1.03
Kas-1	−30.29	−29.24	1.05
An-1	−38.55	−37.46	1.09
Bay-0	−38.51	−37.38	1.13
Kil-0	−37.61	−36.45	1.16
Per-1	−34.51	−33.26	1.25
Rsch-4	−36.78	−35.46	1.32
Te-0	−30.39	−29.03	1.37
Col-0	−36.68	−35.02	1.66
Ler-0	−38.75	−37.04	1.72
Pa-1	−38.94	−37.21	1.73
Lc-0	−37.4	−35.53	1.87
Ws-0	−39.19	−37.22	1.97
Ts-1	−31.52	−29.37	2.14
Van-0	−34.61	−31.58	3.04
Sha	−37.68	−34.47	3.21
Lm-2	−38.7	−34.87	3.83
Ka-0	−38.71	−33.64	5.07

### 3.8 Genetic background

**Figure 6:**
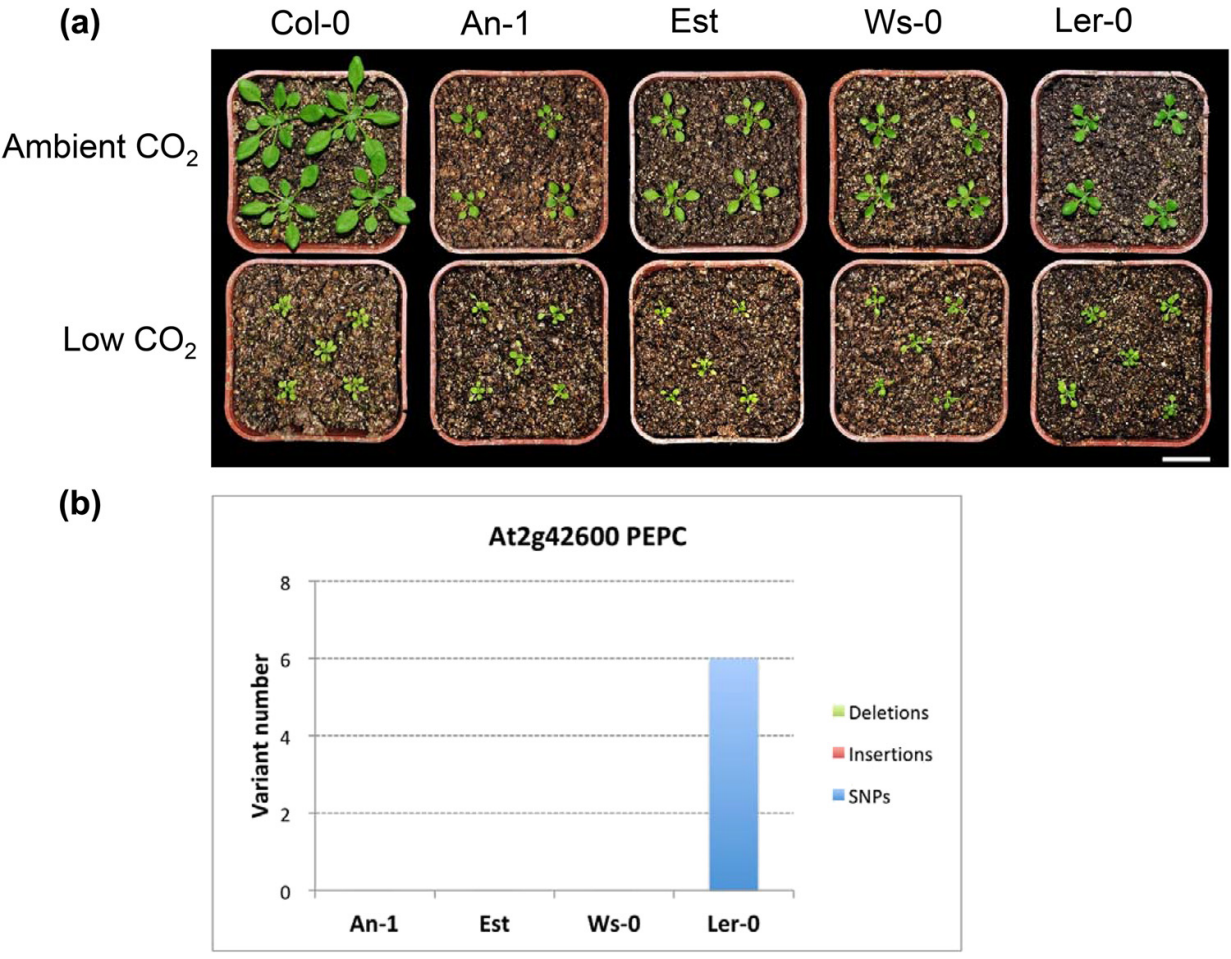
Phenotype and genetic difference of the four accessions An-1, Est, Ws-0, and Ler-0. (a) Phenotype of An-1, Est, Ws-0, and Ler-0 grown in ambient CO_2_ or low CO_2_. Scale bar = 2 cm. (b) The number of polymorphic variants (deletions, insertions, and SNPs) of the C_4_-cycle *PEPC* gene in accessions An-1, Est, Ws-0, and Ler-0 when compared to the Col-0 reference genome. The calculation was performed using the tool POLYMORPH (http://tools.1001genomes.org/polymorph/).

**Figure A2:**
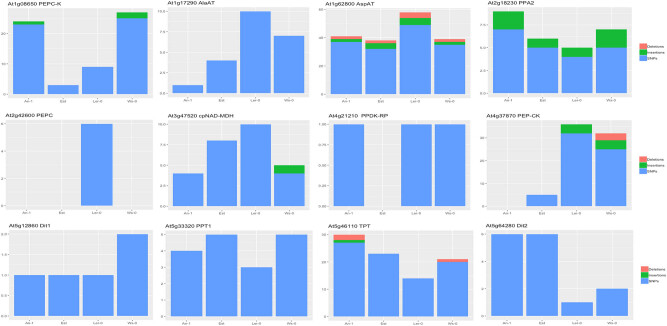
Genetic difference in C_4_-cycle genes and C_4_-related transporter. The number of polymorphic variants (deletions, insertions, and SNPs) of C_4_-cycle genes and C_4_-related transporters in accessions An-1, Est, Ler-0, and Ws-0 when compared to the Col-0 reference genome. The calculation was performed using the tool POLYMORPH (http://tools.1001genomes.org/polymorph/).

From the aforementioned results, the four accessions An-1, Est, Ws-0, and Ler-0 showed less variation in flowering time, shoot biomass, rosette leaf number, and SLW between the two treatments than did Col-0 (Figures 1–3 and 5). Supporting these data, all four of these accessions exhibited smaller overall size compared to Col-0 under ambient CO_2_ (Figure 6A). Our previous research [30] showed that low-CO_2_ treatment upregulated some C_4_-cycle genes including *PEPC* [45] and *PEPC-K* in *Arabidopsis* accession Col-0. The 1001 Genomes Project (https://1001genomes.org) provided whole genome sequence data to interrogate for genetic differences between different accessions, thus allowing us to potentially decipher how phenotypic variation is related to underlying genetic variation. We used the tool POLYMORPH (http://tools.1001genomes.org/polymorph/) to examine if low-CO_2_-responsive genes carried specific sequence changes among the four accessions we screened with the most extreme responses to low CO_2_. We calculated all polymorphic variants, including single nucleotide polymorphisms (SNPs), insertions, and deletions in all C_4_-cycle genes and C_4_-related transporter genes in the An-1, Est, Ws-0, and Ler-0 four accessions. However, no clear pattern in genetic variation emerged to indicate the mechanisms driving these phenotypic responses (Figure A2). For example, although *PEPC* (At2g42600) showed a 2.10-fold higher transcript abundance in Col-0 in response to low CO_2_ [30], An-1, Est, and Ws-0 had no identifiable differences in *PEPC* sequence compared to that of Col-0 (Figure 6b). Responses to low CO_2_ stress involve a complex network of regulatory elements to participate in mitigating damage induced by the stress, and differences in genetic background may potentially trigger unique stress responses among different accessions. Using transcriptomics sequence data, Carlson et al. [46] determined that a significant number of SNPs were absent in two accessions of *Arabidopsis suecica* (a relatively recent allopolyploid species) in the 1,001 genome SNP collection. RNA-seq analysis can effectively identify the genetic variation among these four accessions and we will use this technique in further experiments to explore the genetic basis underpinning plant adaptation to low CO_2_.

## 4 Discussion

Over the evolutionary history of plants, a number of stress-responsive adaptations have arisen to ensure that plants can successfully cope with environmental stresses. In *Arabidopsis*, intraspecific variation has been reported in responses by different lineages to abiotic stresses and shifts in climate conditions [47]. Our objective for the current study was to investigate potentially heritable phenotypic variation in response to low CO_2_ stress in *Arabidopsis*. In this study, the 31 *Arabidopsis* accessions from different geographic regions (Table 1) were selected for comparison of traits related to reproductive fitness and carbon assimilation under low (100 ppm) CO_2_ and ambient (380 ppm) CO_2_, in order to identify the most adaptable accessions under low CO_2_.

### 4.1 *Arabidopsis* showed substantial natural phenotypic variation among wild accessions in response to low CO_2_

Flowering time is an important determinant of plant fitness and represents a discrete developmental transition in response to environmental conditions [26]. Shifts in flowering time in response to low CO_2_ availability have previously been observed for some *Arabidopsis* lines [23,30]. In this study, we observed significant variation in flowering time across accessions. Compared to ambient conditions, 12 accessions, including An-1, Est, Ws-0, and Ler-0, took the same amount of days to reach stage 5.10 under low CO_2_. Only two accessions, Mc-0 and Rsch-4, flowered earlier (by 4 days) under CO_2_ limitation, whereas among the 17 late-flowering *Arabidopsis* accessions, Ts-1 took 54 days more to reach stage 5.10, and Te-0 and Kas-1 died under low CO_2_ without ever flowering (Figure 1a and b). The delay in first flower opening thus ranged from 1 day (Rsch-4) to 63 days (Lip-0). Two other accessions, Est and Ga-0, died after stage 6.00 under low, but not ambient CO_2_.

Rosette leaf number is commonly used as a standard indicator of flowering time in *Arabidopsis* and with late flowering plants typically developing more rosette leaves. In our screen, six accessions An-1, Est, Pa-1, Ws-0, Ler-0, and Sha showed a slight difference in leaf number between treatments, whereas some accessions (Tsu-1, Be-0, Rsch-4, and Mc-0) showed a greater than 50% reduction in rosette leaf number (Figure 3). There is a very strong correlation between the time to flowering and the number of leaves at flowering in *Arabidopsis* [35], with previous study suggesting that these two traits may be genetically linked in wild accessions [48]. In our experiment, low CO_2_ treatment delayed flowering time but did not increase the rosette leaf number, although a logical expectation would be that leaf number continues to increase as the duration of vegetative growth prior to flowering is prolonged. This finding agrees with results reported by Salomé et al. [48] for an F2 population derived from natural accessions in which the traits of “days to flower” and “leaf number” were canalized in natural accessions, though the link between the two could be genetically uncoupled. Taken together, these results suggest that response to low CO_2_ entails a combination of physiological and morphological changes that maximize the efficiency of carbon assimilation in order to maintain consistent reproductive processes.

Changes in CO_2_ concentration can induce profound effects on plant growth because of the central necessity for CO_2_ in plant metabolism. Higher atmospheric CO_2_ concentrations often boost the growth and reproduction of C_3_ annuals, whereas low CO_2_ has the opposite effect and decreases plant growth [30,49]. Previous studies showed that low CO_2_ availability was a limiting factor in plant growth, leading to reduced production of plant biomass [18,23,30,50,51,52,53,54]. However, a delay of first flower opening was common among the 31 accessions under low CO_2_ stress, the biomass of all accessions in our study decreased. Though all plants in this study, regardless of accession, exhibited a dwarfed morphology and decreased biomass when grown under CO_2_-starvation conditions, we observed extensive variation in aboveground biomass, which ranged from 58% to greater than 95% lower biomass compared to their growth at full CO_2_ availability (Figure 2). Furthermore, the accessions that grew the fastest under full, ambient CO_2_ also showed the greatest reduction in biomass at low CO_2_, consistent with previous research [55].

In general, subjecting plants to growth at 100 ppm CO_2_ induced significant changes to vegetative growth and reproductive development across a range of phenotypic traits (Table 2), including later flowering, dwarf stature, reduced biomass, and reduced rosette leaf number, which varied among the *Arabidopsis* accessions. Thus, individual accessions may have developed an adaptive response to low CO_2_ that can be used to further determine the genetic variability responsible for this adaptation.

### 4.2 Altitude of origin did not relate to low-CO_2_ response

The partial pressure of atmospheric CO_2_ decreases with the increase in altitude. *Arabidopsis* accessions adapted to growth at higher altitudes have presumably undergone a stronger selection for growth in lower CO_2_ concentration than that of low altitude accessions. We hypothesized that adaptation to low CO_2_ increases along an altitudinal gradient. To test this hypothesis, we used 31 *Arabidopsis* accessions that were originally collected from a variety of altitudes ranging from 50 to 3,400 m above sea level (Table 1). In Figures 1–5, the *Arabidopsis* accessions listed on the *x*-axis (left to right) are arranged by altitude, in the same order as in Table 1. However, we found that the responses to low CO_2_ for all of the changes in measured traits in this study did not correlate linearly with altitude. For example, although all accessions had significantly lower aboveground biomass at low compared with ambient CO_2_, six accessions An-1 (50 m), Est (50 m), Pa-1 (50 m), Ws-0 (150 m), Ler-0 (600 m), and Sha (3,400 m) performed much better than Col-0 (50 m) (Figure 2), suggesting that there was no clear differentiation between low-altitude genotypes and high-altitude genotypes. Ward and Strain [23] examined the responses to 20 Pa (200 ppm) CO_2_ in eight accessions from seven different altitudes between sea level and 3,400 m and found that accessions exhibited limited heritable variation in the response of biomass production. Therefore, in this work, the altitude of origin did not significantly affect vegetative growth in response to low CO_2_ (100 ppm).

### 4.3 Ws-0 was least affected by low CO_2_

In this study, we found that the accessions An-1, Est, Ws-0, and Ler-0 showed less variation in flowering time, shoot biomass, rosette leaf number, and SLW between the two treatments compared to Col-0 (Figures 1–3 and 5). Compared to other accessions, their flowering time and rosette leaf number remained almost the same in the two CO_2_ treatments, and shoot biomass was significantly less affected than in other accessions. Furthermore, the SLW was less affected by low CO_2_, compared to accession Col-0. In light of the combined quantitative trait data, Est and Ws-0 were the least affected among these four accessions. As mentioned above, Est did not set seeds under low CO_2_ (Figure A1), therefore Ws-0 would be the most effective candidate for further quantitative genetics studies.

When we examined the phenotypes of these four accessions grown under low CO_2_, we found that they did not have a bigger plant size, whereas under ambient CO_2_, they showed a smaller stature compared to other accessions. Temme et al. [55] reported species with fast growth or largest biomass at ambient CO_2_ showed the strongest absolute reduction at low CO_2_. Nitrogen content and photosynthetic rate are strongly affected by low CO_2_ [18,56,57]. Previous studies have proposed that stress-tolerant plants have lower growth rates (reviewed in ref. [58]). One explanation of our observations is that their smaller stature and relatively slow growth under ambient CO_2_ is an advantage in response to low CO_2_. The small stature or slow growth among some plants may indicate low energy and low carbon demands, thus C–N cycle and photosynthesis may be less affected and these plants show less affect when grown under low CO_2_. If this hypothesis is correct, it can provide us with valuable insight into the mechanisms by which C_4_ metabolism arose and the reasons why it evolved independently in grasses (i.e., roughly half of the known C4 species are grasses) and also in a number of eudicots, for example, *Amaranthaceae*, *Euphorbiaceae*, *Asteraceae*, and *Boraginaceae* [59].

In this study, we compared the phenotypic variation in response to low (100 ppm) CO_2_ among 31 *Arabidopsis* accessions to assess their relative adaptability through sustained vegetative growth and reproductive development. We found that *A. thaliana* displays extensive variation in its ability to adapt to low CO_2_ and that this variation was correlated with their rate of growth under non-CO_2_-limited conditions, rather than the altitude of origin for individual accessions. In particular, accession Ws-0 showed the least variability between treatments, indicating that it was the best potential candidate for use in further quantitative genetics studies and for isolation of genes underlying low CO_2_ response. We also propose that a lower growth rate can attenuate the effects of low-CO_2_ stress, though further experimental evidence is needed to test this hypothesis. Our findings on the physiological effects of growth under low CO_2_ provide insight into the mechanisms by which individual plants and whole ecosystems may adapt to changes in atmospheric CO_2_. As atmospheric levels of CO_2_ rise, our increased understanding of these mechanisms governing carbon assimilation and flowering time during stress can improve our capability to predict the future of natural ecosystems subject to increasingly wide variations in climate.
